# Comparison of Functional Outcomes of an Anterior Cruciate Ligament (ACL) Reconstruction Using a Peroneus Longus Graft as an Alternative to the Hamstring Tendon Graft

**DOI:** 10.7759/cureus.37273

**Published:** 2023-04-07

**Authors:** Abhishek Agarwal, Shitanshu Singh, Arpit Singh, Prakash Tewari

**Affiliations:** 1 Sports Medicine, King George's Medical University, Lucknow, IND; 2 Orthopaedic Surgery, King George's Medical University, Lucknow, IND

**Keywords:** hamstring tendon grafts, hamstring tendon, peroneus longus tendon graft, road traffic accident, american orthopaedic foot and ankle society, bone-patellar tendon-bone (bptb), hamstring tendons (semitendinosus and gracilis), anterior cruciate ligament reconstruction, anterior cruciate ligament

## Abstract

Background

Ever since the arthroscopic reconstruction of the anterior cruciate ligament (ACL) has begun, the use of the peroneus longus (PL) graft for primary ACL reconstruction (ACLR) has never been thought of. There is very little literature on it. Hence, our study aims to compare the functional outcomes, knee stability, donor site morbidity, and assessment of thigh muscle wasting in patients with ACL injury managed by arthroscopic single bundle reconstruction with peroneus longus tendon (PLT) and hamstring tendons (HT), respectively.

Methods

All adults aged 16-50 years of either gender presenting with symptoms of symptomatic ACL deficiency were admitted for arthroscopic single-bundle ACLR and allocated into two groups (peroneus longus and hamstring tendon). Functional scores (International Knee Documentation Committee (IKDC), Lysholm score), clinical knee evaluation (anterior drawer, Lachman, and pivot shift test), donor site morbidity (American Orthopedic Foot and Ankle Society ankle hindfoot score (AOFAS)), and thigh circumference were recorded preoperatively and at six months and one year postoperatively. The same post-op rehabilitation protocol was followed in both groups.

Results

One hundred and ninety-four patients (hamstring n = 96, peroneus n = 98) met the inclusion criteria. There were no significant differences between the preoperative, six-month postoperative, and one-year postoperative scores between the hamstring and peroneus longus groups in the IKDC (p=0.356) and Lysholm knee score (p=0.289). The mean for the AOFAS was 99.05 ± 3.56 and 99.80 ± 0.70 in the PLT and HT groups, respectively, showing no statistical difference, with a significant improvement in thigh muscle wasting among the PLT group at final follow-up (p < 0.001).

Conclusion

We observed similar knee stability and functional outcomes and no obvious donor site morbidity among both groups. These patients also had better responses to physiotherapy in recovering from their thigh muscle wasting. So, we can recommend that a PL graft can be a safe, viable, and effective option for usual arthroscopic single-bundle ACL reconstruction.

## Introduction

The anterior cruciate ligament (ACL) is the most frequently injured knee joint ligament [1, 2], and hence its satisfactory reconstruction is a matter of utmost importance. The management of ACL deficiency by anterior cruciate ligament reconstruction (ACLR) using the patient's own tendon (autograft) is an extensively performed procedure [2]. The most commonly utilized autografts comprise the hamstring tendons (semitendinosus and gracilis) [3], the bone-patellar tendon-bone (BPTB) [4], and the quadriceps tendon [5]. As evidenced by the most recent studies, BPTB is the graft of choice since it has the property of bone-to-bone healing, which permits the effective fusion of grafts with the tunnels, leading to a quick return to the patient's professional activities. This distinctive feature is important, especially among professional athletes with ACL injuries. However, it carries the risk of patellar fracture (especially in the Asian population) [6], fat pad fibrosis [7], and patellar tendon contracture [8] with an invasive approach owing to a relatively longer incision, fixed-length graft, and weaker tensile strength than native ACL, making it undesirable for usual reconstruction where pain-free kneeling is also crucial, especially during some religious practices. Therefore, hamstring tendons have now become the preferred graft, as they are relatively easier to harvest, have minimal donor site morbidity, and have a tensile strength comparable to that of the native ACL. But unpredictable graft size (leading to a scuffle in the surgeon’s mind to use plastic fiber tape augmentation), risk of saphenous nerve paresthesia, and a potential decrease in hamstring muscle strength [9], which is crucial for some athletes who need dominant hamstring power, add to its demerits. Optimal hamstring strength is crucial for newly reconstructed ACL patients as it counters anterior translation of the tibia by quadriceps muscle contraction [10] and prevents quadriceps hamstring asymmetry.

Due to the functional limitations of these autografts, surgeons are in constant search of an ideal autograft that is easy to harvest with minimum donor site morbidity and could be used in patients of every ethnicity without compromising their activities of daily living. Recently, the peroneus longus tendon (PLT) for regular ACLR has been under investigation as a potential graft of choice. There is also no risk of post-op hamstring muscle weakness or injury to the saphenous nerve during graft retrieval. As the PLT has good biomechanical properties and a high load-to-failure strength [11], its use as a graft for ACLR is prevailing among orthopedic surgeons [12]. Studies have reported that the peroneus brevis tendon (PBT) is a more competent ankle evertor [13], justifying the harvest of PLT as far as ankle evertor functions are concerned. Moreover, PL has already been used for other ligament reconstructions and cruciate ligament reconstructions in multiligamentous injuries. To our knowledge, no comparative study exists between the use of PLT and HT in primary ACLR. Therefore, this study aims to compare the functional outcome of arthroscopic anterior cruciate ligament reconstruction with hamstring tendon versus peroneus longus tendon and to evaluate knee stability, donor site morbidity, and improvement in thigh wasting in patients with an ACL injury.

## Materials and methods

**Figure 1:**
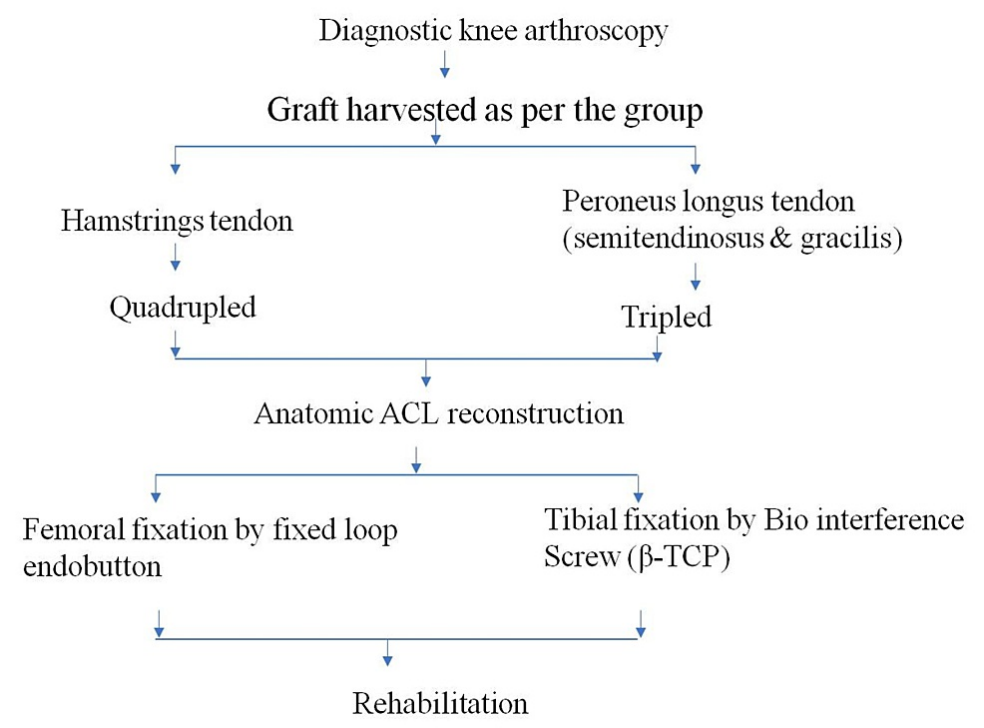
A flowchart of the procedure and arthroscopic technique

This prospective cohort study was conducted at King George’s Medical University, a tertiary care centre in Lucknow, India, after obtaining formal approval from the institute's ethical committee (ref. code: 102 ECM II B - Thesis /P 114). This study was done in June 2020-December 2021, with 194 patients with ages between 16 and 50 years of either gender who presented with an isolated symptomatic ACL rupture being included after proper written informed consent. Patients were excluded if they had other associated intraarticular pathology, stiffness of the joint, multiligamentous injury, or associated fractures around the knee joint. All included patients were randomly allocated into HT and PLT groups, operated on, and then rehabilitated. For both groups, patients were made to fill out the International Knee Documentation Committee (IKDC) score form, the Lysholm score form, and the ankle-hindfoot score form at the preoperative stage and during subsequent follow-up periods. The thigh circumference of the injured limb was measured 15 cm proximal to the superior pole of the patella and compared with the contralateral healthy side. Similarly, all the patients were also assessed for knee stability using clinical tests (the anterior drawer, Lachman, and pivot shift tests). The procedure and arthroscopic technique are shown in Figure 1.

Rehabilitation Protocol

Both groups were treated with the same rehabilitation protocol as shown in Table 1.

**Table 1:** Rehabilitation protocol for Isolated single-bundle ACL reconstruction

Timeline	Rehabilitation protocol
Day 1- Day 7 post surgery	Ice fomentation (two to three times a day), anti-inflammatory pain killers, a knee brace for static close-chain quadriceps exercises, full passive extension close-chain hamstring exercises, partial weight-bearing (up to 30%) with the help of a walker (with the knee brace locked in full extension), check dressing on day three
Week 1 to Week 2	Stitch removal (days 10–14), full passive extension, anti-inflammatory pain killers, a knee brace on static close-chain quadriceps exercises, passive close-chain knee bending exercises (up to 60 degrees), close-chain hamstring exercises, and partial weight bearing (up to 50%) with the help of a stick (with the knee brace locked in full extension)
Week 2 to Week 4	Open-chain quadriceps exercise (short of 20 degrees), active open-chain knee bending exercises (up to 90 degrees), hamstring exercises along gradual full weight bearing (with the knee brace unlocked)
Month 1 to Month 3	Quadriceps and hamstring exercises are to be continued. Full weight bearing without any brace.
Month 3 to Month 4	Resistance exercises (as per comfort), supported partial squats
Month 4 to Month 6	Resistance exercises (as per comfort), unsupported partial squats, jogging (five to 10 minutes)
Month 6 to Month 8	No pivoting, non-contact sports (as per the strength of muscles and stability)
Month 8 to Month 10	Pivoting, non-contact sports (as per the strength of muscles and stability)
Beyond 10 Months	The patient can resume all types of sports

## Results

During the study period, 194 patients who fulfilled the inclusion criteria underwent an isolated single-bundle ACL reconstruction. The demographic data of the study population were analysed as shown in Table 2, and the mode of injury and occupation of the observed population was assessed as shown in Table 3.

**Table 2:** Demographic data of the study population

Variable	Peroneus longus tendon	Hamstring tendon	p-value
Age	28.00 ± 4.91	27.50 ± 4.06	0.546
Gender (male/female)	68/30	57/39	0.191
Duration from injury to intervention (in months)	15.10 ± 6.23	14.70 ± 6.68	0.667

**Table 3:** Mode of injuries

Mode of injuries	Occupation	Peroneus longus graft group (n=98)	Hamstring tendon graft group (n=96)	Chi-square	p-value
		n	n		
Contact sports injury	Student	12	9	4.94	0.085
	Sportsperson	9	6		
	Other	0	4		
Non-contact sports injury	Student	9	10	3.41	0.182
	Sportsperson	5	19		
	Other	5	9		
Road traffic accident	Student	18	15	0.29	0.590
	Sportsperson	0	0		
	Other	40	24		

Assessment of Knee Stability Among Both the Groups

The knee stability was assessed using the anterior drawer test, Lachman test, and pivot shift test. This is shown in Table 4 and Table 5, respectively.

**Table 4:** Knee stability was assessed using the anterior drawer test, the Lachman test, and the pivot shift test

	Peroneus longus graft group (n=98)		Hamstring tendon graft group (n=96)		Chi-square	p-value
	n	%	n	%		
Anterior drawer test						
Pre-op						
Grade 0	0	0.00	0	0.00	0.07	0.795
Grade 1	0	0.00	0	0.00		
Grade 2	14	14.29	16	16.67		
Grade 3	84	85.71	80	83.33		
Six months		0.00		0.00		
Grade 0	94	95.92	89	92.71	0.93	0.334
Grade 1	4	4.08	7	7.29		
Grade 2	0	0.00	0	0.00		
Grade 3	0	0.00	0	0.00		
One year		0.00		0.00		
Grade 0	95	96.94	92	95.83	1.69	0.429
Grade 1	2	2.04	4	4.17		
Grade 2	0	0.00	0	0.00		
Grade 3	1	1.02	0	0.00		
Lachman test						

Pre-op						
Grade 0	0	0.00	0	0.00	2.87	0.090
Grade 1	0	0.00	0	0.00		
Grade 2	10	10.20	18	18.75		
Grade 3	88	89.80	78	81.25		
Six months						
Grade 0	90	91.84	85	88.54	0.60	0.440
Grade 1	8	8.16	11	11.46		
Grade 2	0	0.00	0	0.00		
Grade 3	0	0.00	0	0.00		
One year						
Grade 0	91	92.86	86	89.58	2.12	0.346
Grade 1	6	6.12	10	10.42		
Grade 2	0	0.00	0	0.00		
Grade 3	1	1.02	0	0.00		

**Table 5:** Pivot shift test

	Peroneus longus graft group (n=98)		Hamstring tendon graft group (n=96)	
	Positive	Negative	Positive	Negative
Pre-op	98	0	96	0
Six months	0	98	0	96
One year	1	97	0	96

Based on the anterior drawer test, a total of 187 patients among both groups achieved a negative anterior drawer test at the one-year postoperative period, while six patients had a grade 1 score. One patient in the PLT group reported a grade 3 anterior drawer test due to re-injury. As per the Lachman test, 177 patients in our study were evaluated to have a negative test at final follow-up, while a total of 16 patients had a grade 1 score. One patient in the PLT group who reported a grade 3 anterior drawer test also had a grade 3 Lachman test.

All patients in our study reported a negative pivot shift test except one patient in the PLT group who had an incident of a road travel accident (RTA), which was a re-injury, after a six-month follow-up period and still complained of knee instability in his daily life at the one-year follow-up.

Functional Outcome of Patients

The functional outcome of patients was assessed using the International Knee Documentation Committee (IKDC) and Lysholm knee score, whose results are shown in Table 6. The mean IKDC score at six-month and one-year follow-ups was reported to be 83.28 and 94.13, respectively, among the PLT group and 79.73 and 95.12, respectively, among the HT group. The mean Lysholm knee score among the PLT group was found to be 97.00 and 99.15 at six-month and one-year follow-ups, respectively, while among the HT group, it was 96.35 and 99.85 at their respective follow-ups.

Donor Site Morbidity 

It was assessed using the American Orthopaedic Foot and Ankle Society score (AOFAS), which was reported to be 96.2 and 96.0 in PLT and HT groups, respectively, and both were significantly increased in post-op periods to 99.05 and 99.80, respectively. We reported no statistical difference between both (Table 6).

The Difference in Thigh Circumference Between the Operated and Sound Leg

The mean changes in thigh circumference of the injured side (in cm) from the uninjured side were analysed and noted; there was a significant increase in thigh circumference among the PLT group with a difference of 1.72 cm from the uninjured side at preoperative state to 0.714 cm and 0.216 cm, respectively, at six months and one-year follow-up, as compared to the HT group with a difference of 1.93 cm from the normal side at the preoperative period to 1.37 cm and 0.88 cm, respectively, at six months and one-year follow-up, as shown in Table 6.

**Table 6:** The functional outcome of patients was assessed using the IKDC (International knee documentation committee) and Lysholm knee score

	Peroneus longus graft group (n=98)		Hamstring tendon graft group (n=96)		t-test	p-value
	Mean	±SD	Mean	±SD		
Lysholm knee score						
Pre-op	51.35	29.22	57.05	25.23	-0.66	0.513
Six months	97.00	0.00	96.35	1.60	1.82	0.077
One year	99.15	2.89	99.85	0.37	-1.08	0.289
IKDC score						
Pre-op	53.62	3.65	52.52	3.28	2.15	0.238
Six months	83.28	3.71	79.73	6.83	2.04	0.058
One year	94.13	4.66	95.12	0.73	-0.93	0.356
Ankle hindfoot score						
Pre-op	96.20	0.95	96.00	1.03	0.639	0.527
Six months	99.75	0.44	99.72	0.34	-0.517	0.516
One year	99.05	3.56	99.80	0.70	-0.924	0.361
The mean difference in thigh muscle circumference (uninjured side-injured side)						
Pre-op	1.72	0.670	1.93	0.57	-1.073	0.289
Six months	0.714	0.505	1.37	0.53	-2.583	0.013
One year	0.216	0.451	0.88	0.54	-4.199	<0.001

## Discussion

Through our study, it was found that the activity level at one year was close to the pre-injury level, with no incidence of re-ruptures at subsequent follow-ups.

Previous studies have demonstrated excellent knee stability in ACL-deficient patients with both PLT and HT grafts. We assessed the stability using the anterior drawer, Lachman, and pivot shift tests. Lachman and the anterior drawer test assessed the forward translation of the tibial plateau with respect to the distal femur. There were significant improvements in both groups, and they were comparable at every follow-up period with no statistically significant difference. Our studies had similar findings to those of Rhatomy, S et al., who also reported comparable anterior drawer and Lachman test results between both groups [14]. Trung et al. evaluated the rate of the negative anterior drawer test to be 96.7%; level one was 3.3% and was no longer level two and three; and the rate of the Lachman test to be 90%; level one was 10% and was no longer level two and three [15].

The pivot shift test was used to assess the rotational stability of the knee. In our study, only one patient in the PLT group reported a positive test postoperatively, which was due to reinjury. Other studies, like Trung et al.'s, who used PLT grafts, reported a negative pivot shift among 93.3% of patients; level one was 6.7%; levels two and three were no longer reported [15]. Kumar VK et al. reported a negative rate of 96%; level one was 4%; and levels two and three were no longer reported [16].

Functional outcomes of patients among both groups were found to be equivalent at every follow-up, with a mean Lysholm knee score of 99.15 among the PLT group and 99.85 among HT patients at one-year follow-up and a mean IKDC score of 94.13 and 95.12 in patients of the PLT and HT groups, respectively. At the end of the follow-up, no patients in either group had flexion or extension loss. Studies like Rhatomy S et al. also reported an insignificant difference in context to the IKDC and Lysholm knee score between the two groups [17]. He, J., Tang et al. reported a better Lysholm knee score among the PLT group [18].

Donor site morbidity of the ankle was evaluated using the ankle-hindfoot score, which was found to be insignificantly different between the two groups with mean scores of 99.05 and 99.80 in PLT and HT group patients, respectively, at one-year follow-up. The primary concern of the donor site (ankle) was the deficit of eversion while the patient is in the stance phase of gait and ankle instability [19]. None of our patients had such complaints. This can be attributed to the dominance of peroneus brevis in ankle eversion and the regeneration potential of the harvested graft. This infers that harvesting the peroneus longus tendon does not cause any ankle morbidity, and its resection has no major influence on the ankle joint [20].

We measured the thigh circumference on the injured side at pre-op and compared it with that of the uninjured side. The patients in the PLT group showed better improvement in thigh muscle hypotrophy as compared to their pre-op status, with a mean difference in thigh circumference of 0.216 cm at the final follow-up from 1.72 cm (the difference at pre-op), whereas the HT group had a mean difference of 0.881 cm from 1.93 cm (the difference at pre-op), respectively. Similar results were seen by Rhatomy S et al., where significantly less thigh hypotrophy was noted among the PLT group at one year of follow-up [17]. Thigh hypotrophy due to hamstring (semitendinosus and gracilis) tendon harvest results in decreased strength, resulting in quadriceps-hamstring asymmetry, which results in an imbalance in dynamic knee stability [20]. So it's evident that the patients in the PLT group had better post-op recovery with significantly improved thigh wasting.

## Conclusions

Our study concludes that the patients among whom the peroneus longus tendon was used as an autograft for arthroscopic single-bundle ACL reconstruction had an excellent functional outcome (based on IKDC and Lysholm knee scores) and demonstrated similar post-op knee stability (based on the anterior drawer, Lachman, and pivot shift test scores) to a quadruple hamstring tendon graft. It had no significant donor site morbidity and better improvement of thigh muscle wasting (a superior response to rehabilitation to that of the HT group), making it an effective and safe autograft option for usual ACL reconstruction.
